# Genome-wide identification and expression analysis of the *ClTCP* transcription factors in *Citrullus lanatus*

**DOI:** 10.1186/s12870-016-0765-9

**Published:** 2016-04-12

**Authors:** Pibiao Shi, Kateta Malangisha Guy, Weifang Wu, Bingsheng Fang, Jinghua Yang, Mingfang Zhang, Zhongyuan Hu

**Affiliations:** Laboratory of Germplasm Innovation and Molecular Breeding, Institute of Vegetable Science, Zhejiang University, Hangzhou, 310058 P.R. China; Key laboratory of Horticultural Plant Growth, Development & Quality Improvement, Ministry of Agriculture, Hangzhou, 310058 P.R. China; Zhejiang Provincial Key Laboratory of Horticultural Plant Integrative Biology, Hangzhou, 310058 P.R. China; Faculty of Agronomy, Lubumbashi University, Lubumbashi, D.R. Congo

**Keywords:** TCP, Transcription factors, Watermelon, Internode elongation

## Abstract

**Background:**

The plant-specific TCP transcription factor family, which is involved in the regulation of cell growth and proliferation, performs diverse functions in multiple aspects of plant growth and development. However, no comprehensive analysis of the TCP family in watermelon (*Citrullus lanatus*) has been undertaken previously.

**Results:**

A total of 27 watermelon TCP encoding genes distributed on nine chromosomes were identified. Phylogenetic analysis clustered the genes into 11 distinct subgroups. Furthermore, phylogenetic and structural analyses distinguished two homology classes within the ClTCP family, designated Class I and Class II. The Class II genes were differentiated into two subclasses, the CIN subclass and the CYC/TB1 subclass. The expression patterns of all members were determined by semi-quantitative PCR. The functions of two *ClTCP* genes, *ClTCP14a* and *ClTCP*15, in regulating plant height were confirmed by ectopic expression in *Arabidopsis* wild-type and ortholog mutants.

**Conclusions:**

This study represents the first genome-wide analysis of the watermelon TCP gene family, which provides valuable information for understanding the classification and functions of the TCP genes in watermelon.

**Electronic supplementary material:**

The online version of this article (doi:10.1186/s12870-016-0765-9) contains supplementary material, which is available to authorized users.

## Background

The TCP gene family, a small group of transcription factors (TF) exclusive to higher plants, was first described in 1999 [1]. The family plays important roles in regulating diverse physiological and biological processes, including phytohormone biosynthesis and signal transduction, leaf morphogenesis and senescence, branching, flower development, pollen development and the circadian clock [2–15]. TCP proteins are characterized by a 59-amino-acid non-canonical basic-Helix-Loop-Helix (bHLH) motif that is responsible for DNA binding, nuclear targeting and pair-wise protein–protein interaction [1, 16]. This domain was first identified from four proteins with critical roles in the evolution and developmental control of plant morphology: TEOSINTE BRANCHED 1 (TB1) of maize (*Zea mays*), CYCLOIDEA (CYC) of snapdragon (*Antirrhinum majus*) and the PROLIFERATING CELL FACTORS 1 and 2 (PCF1 and PCF2) of rice (*Oryza sativa*) [16–18]. Thus the name of the TCP TF family is derived from the acronym for these proteins. TCP genes can be divided into two subfamilies based on the homology of the TCP domains: class I (or TCP-P) and class II (or TCP-C) [19]. TCP class I, also known as the PCF subfamily, contains rice OsPCF1 and OsPCF2, whereas TCP class II is further subdivided into the CIN and CYC/TB1 subclades [7]. The most obvious difference between the two classes is a four-amino-acid deletion in the basic region of the TCP domain of class I compared with that of class II proteins. Moreover, the DNA binding sequence for the two classes differs slightly but partly overlaps (GGNCCCAC for class I and GTGGNCCC for class II) [20, 21].

Accumulating evidence confirms that class I TCP proteins mainly play a role in cell growth and proliferation [13, 20], whereas the CIN proteins may be involved in lateral organ development and the CYC/TB1 clade is mainly involved in the development of axillary meristems giving rise to either flowers or lateral shoots [5, 7, 9, 22–27]. Generally, the two classes of TCP genes are considered to act antagonistically on specific biological processes. Class I genes are usually assumed to promote plant growth, mainly based on the finding that OsPCF1/OsPCF2 and AtTCP20 act as transcriptional activators of *PCNA* and *CYCB1;1* genes [7, 20, 28]. In practice, most class I single mutants do not show conspicuous phenotypic variation, which might be because of functional redundancies. For example, increasing evidence demonstrates that *AtTCP14* and *AtTCP15* function redundantly to regulate biological processes and influence plant structure. The two genes also mediate responses of leaves and flowers to cytokinin and promotion of seed germination by gibberellin (GA) [29–31]. More recently, *AtTCP14* and *AtTCP15* were shown to repress endoreduplication by directly regulating the expression of cell-cycle genes to influence cell and organ growth [32]. Notable plant morphological changes are observed in the *tcp14 tcp15* double mutant, such as shortened internode length as well as varied leaf and sepal morphology, whereas single mutants show mild phenotypic defects [29, 33]. Moreover, *AtTCP9* and *AtTCP19* play a positive role in a redundant manner with *AtTCP20* in the control of leaf senescence, as *tcp9 tcp20* and *tcp19 tcp20* double mutants exhibit earlier onset of senescence in comparison with the wild type, whereas none of the single mutants exhibit accelerated senescence [13, 15].

By contrast, many phenotypic observations on mutants suggest that the class II TCP proteins usually have preventative roles in cell growth and proliferation. CIN-type genes limit cell proliferation at the margins of the developing leaf primordium. In snapdragon, *Arabidopsis* and tomato *cin*-type mutants, leaf cells exhibit the ability to continue to divide for a longer period compared with the wild type, thus generating larger leaves of altered shape and/or with a crinkled surface [2, 21, 25, 34, 35]. In addition, many TB1-type TCP genes act as axillary bud-specific regulators, such as *TB1* of maize [18, 22], *AtBRC1* and *AtBRC2* of *Arabidopsis* [4, 36], *PsBRC1* of pea (*Pisum sativum*) [37] and *OsFC1/OsTB1* of rice [38, 39]. Defects in these genes result in excessive shoot branching, which are indicative of a negative function of these TCP genes on bud activity [4, 36–39]. In some instances, class II TCP genes may also play positive roles in plant growth and development. *AtTCP1*, a CYC/TB1 subclade member, is implicated in the control of floral symmetry [40]. Over-expression of a dominant-negative form of *TCP1*, *TCP1-SRDX*, results in a dwarfed phenotype as well as defects in the longitudinal elongation of cotyledonary petioles, rosette leaves and inflorescence stems in *Arabidopsis* [9, 40].

To date, only a small number of TCP TFs have been identified and functionally characterized in model plants such as *Arabidopsis* and rice. Watermelon (*Citrullus lanatus* L.), an important cucurbit crop, is widely cultivated throughout the world. However, little information is available on the watermelon TCP family. In this study, a global analysis of the TCP gene family in watermelon was carried out for the first time. Twenty-seven *ClTCP* genes were identified in the watermelon genome and a systematic analysis, including determination of chromosomal location, phylogenetic relationships, gene duplication, conserved motifs and expression pattern was performed. Plant height is an important agronomic trait of watermelon. Normally, watermelon genotypes of reduced plant height are more suitable for intensive culture and early maturation in a greenhouse. *ClTCP* genes involved in the regulation of plant height in watermelon were identified in this research.

## Results and discussion

### Identification of TCP genes in *Citrullus lanatus*

**Fig. 1:**
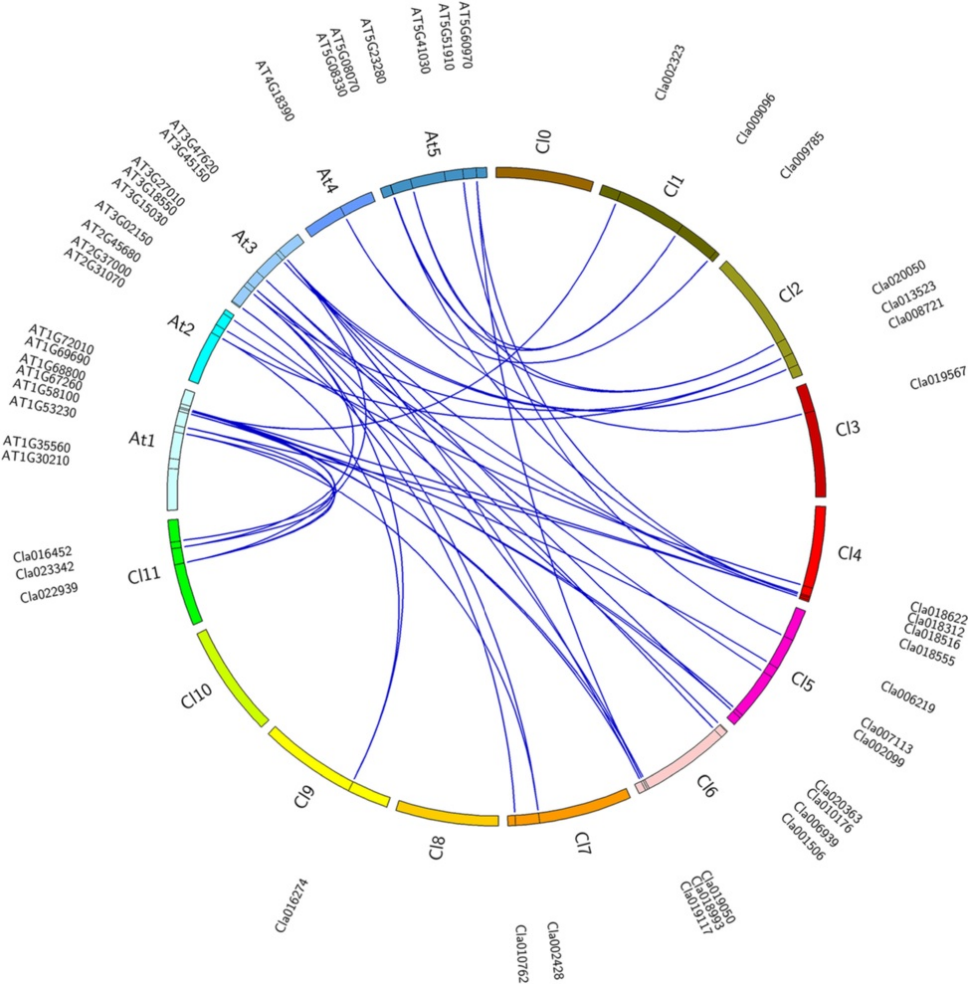
Visualization of the TCP Maps linkage groups. A Circos diagram illustrates the relative positions of TCP genes. The genes are plotted against their linked counterpart chromosomes. Chromosomal locations were determined according to chromosomal location information gathered from TAIR (https://www.arabidopsis.org) and Cucurbit Genomics Database (http://www.icugi.org). The map was obtained using Circos software

To identify TCP protein-coding genes in watermelon, *Arabidopsis* and rice TCP proteins sequences were employed as the query for a BLAST search against the Cucurbit Genomics Database (http://www.icugi.org/cgi-bin/ICuGI/index.cgi). Twenty-seven putative TCP TFs, which contained the conserved TCP domain, were identified (Table 1). The results of a search for watermelon TCP family members in the Plant Transcription Factor Datebase (PlantTFDB; http://planttfdb.cbi.pku.edu.cn) were in agreement with the former search. Due to the lack of standard annotations designated to the 27 TCP genes in watermelon, we named the genes *ClTCP1a* to *ClTCP21* consistent with the *Arabidopsis* TCP proteins that showed the highest sequence similarity and following the gene nomenclature system applied to *Arabidopsis*. The length of the 27 newly identified ClTCP TFs ranged from 182 to 517 amino acids with an average of 332.8 amino acids. Other characteristics of the ClTCP TFs, including molecular weight (Mw), isoelectric point (pI), type and chromosome location, are listed in Table 1. The ClTCP TFs can be classified into the two TCP classes based on the differences within their TCP domains: 12 of the TFs belong to Class I because of the presence of a four-amino-acid deletion in the basic domain relative to the other TFs; the 15 Class II ClTCP TFs can be further clustered into the CIN subclass and the CYC/TB1 subclass (Additional file 1: Figure S1). The genomic location of each *ClTCP* in watermelon is shown in Fig. 1. The 27 ClTCP genes were mapped to nine chromosomes. Moreover, based on a neighbor-joining (NJ) phylogenetic tree constructed from the full-length amino acid sequences, a putative orthologous relationship between the 27 ClTCP TFs and 24 AtTCP TFs was established (Fig. 1 and Additional file 2: Table S1). The number of TCP genes in watermelon is similar to that in *Arabidopsis*, which is in strong agreement with the fact that the number of protein-coding genes in the watermelon genome (23,440 genes) [41] approximates that in *Arabidopsis* (25,498 genes) [42]. A number of *Arabidopsis* TCP genes have more than one counterpart in the watermelon genome, which might be a result of differential gene expansion in watermelon and *Arabidopsis* after their divergence from a common ancestor.

**Table 1:** TCP gene family in *Citrullus lanatus*

Name	Identifier	Length	MW	PI	Type	Chr. Location
		(aa)	(Da)			
*ClTCP1a*	Cla016452	372	41900.2	9.2502	CYC/TB1	Chr11:21507938-21509176
*ClTCP1b*	Cla007113	391	43810.3	9.3686	CYC/TB1	Chr05:15872127-15873302
*ClTCP2a*	Cla009785	453	49073	8.9768	CIN	Chr01:32896256-32897617
*ClTCP2b*	Cla019567	473	51428.6	7.4337	CIN	Chr03:7077952-7079373
*ClTCP3*	Cla023342	438	47583.7	6.2952	CIN	Chr11:19833922-19835238
*ClTCP4*	Cla002428	432	46917.3	6.9235	CIN	Chr07:23567484-23568782
*ClTCP5*	Cla019050	361	39578.9	8.5739	CIN	Chr06:24664979-24666064
*ClTCP7*	Cla009096	264	27651.8	10.4014	PCF	Chr01:22327174-22327968
*ClTCP8*	Cla022939	517	53272.4	7.5207	PCF	Chr11:15780378-15781931
*ClTCP9*	Cla006939	389	41942.8	9.9295	PCF	Chr06:180401-181570
*ClTCP10a*	Cla013523	319	34941.8	8.0631	CIN	Chr02:28431330-28432289
*ClTCP10b*	Cla016274	286	31374	9.3142	CIN	Chr09:10452307-10453167
*ClTCP11*	Cla018312	206	22074.9	8.0236	PCF	Chr04:20715863-20716483
*ClTCP12a*	Cla020363	359	41067.2	9.3598	CYC/TB1	Chr05:30498979-30500058
*ClTCP12b*	Cla002323	241	28002.1	10.0495	CYC/TB1	Chr01:4771786-4772872
*ClTCP13*	Cla010762	300	33416.1	8.7256	CIN	Chr07:29613042-29613944
*ClTCP14a*	Cla019117	398	42508.6	7.7033	PCF	Chr06:25224480-25225676
*ClTCP14b*	Cla018622	283	30621.1	9.3136	PCF	Chr04:23795732-23797562
*ClTCP15*	Cla010176	353	37417.4	9.7333	PCF	Chr05:31441525-31442586
*ClTCP16*	Cla008721	182	20185.6	5.3317	PCF	Chr02:31590819-31591367
*ClTCP17*	Cla018555	209	23699.4	9.5856	CIN	Chr04:23273928-23274557
*ClTCP18a*	Cla018516	235	26945.5	10.6516	CYC/TB1	Chr04:22871510-22872505
*ClTCP18b*	Cla018993	326	37626.9	8.6485	CYC/TB1	Chr06:24195608-24196588
*ClTCP19*	Cla006219	345	36656.2	8.4939	PCF	Chr05:7945454-7946491
*ClTCP20a*	Cla002099	312	33447.1	6.5662	PCF	Chr05:18535808-18536746
*ClTCP20b*	Cla001506	285	30795.3	9.1123	PCF	Chr06:2011967-2012824
*ClTCP21*	Cla020050	257	26673.7	10.5046	PCF	Chr02:24455585-24456358

*aa* amino acid, *MW* molecular weight, *PI* isoelectric point, *Chr* chromosome

### Phylogenetic analysis and conserved motifs

**Fig. 2:**
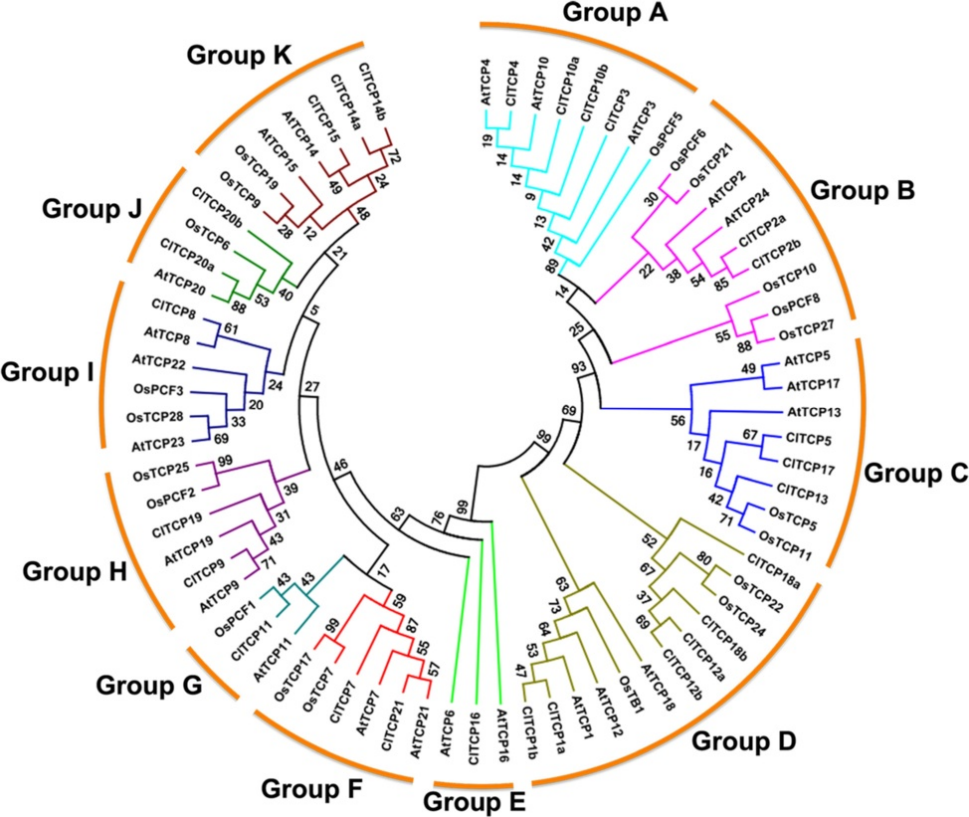
Phylogenetic relationships of TCP transcription factors from watermelon, *Arabidopsis* and rice. The unrooted phylogenetic tree was constructed using MEGA 5.0 with the neighbor-joining method. Support for the topology was assessed by means of a bootstrap analysis with 1000 replicates

To evaluate the phylogenetic relationships among the TCP proteins in watermelon, *Arabidopsis* and rice, an unrooted phylogenetic tree was constructed using the NJ method from a multiple sequence alignment of 27 watermelon, 24 *Arabidopsis* and 21 rice TCP proteins (Fig. 2). The TCPs were divided into 11 subgroups, designated Group A to Group K, according to their sequence features within and outside the TCP domain. The TCPs in Groups A, B and C belonged to the Class II subfamily CIN-type, Group D belonged to the Class II CYC/TB1-type, whereas the remainder of the TCPs belonged to the Class I subfamily (Fig. 2). The TCP genes from the three species were distributed in almost all clades, which indicated that the TCP family diversified before the divergence of these plants. Notably, rice TCP was absent in Group E and a similar result was observed for *Sorghum bicolor* (Fig. 2 and Additional file 3: Figure S2). This finding implies that this clade may have been lost in rice and sorghum, or was acquired in an ancestor of *Arabidopsis* and *Citrullus* after the divergence of monocots and dicots.

**Fig. 3:**
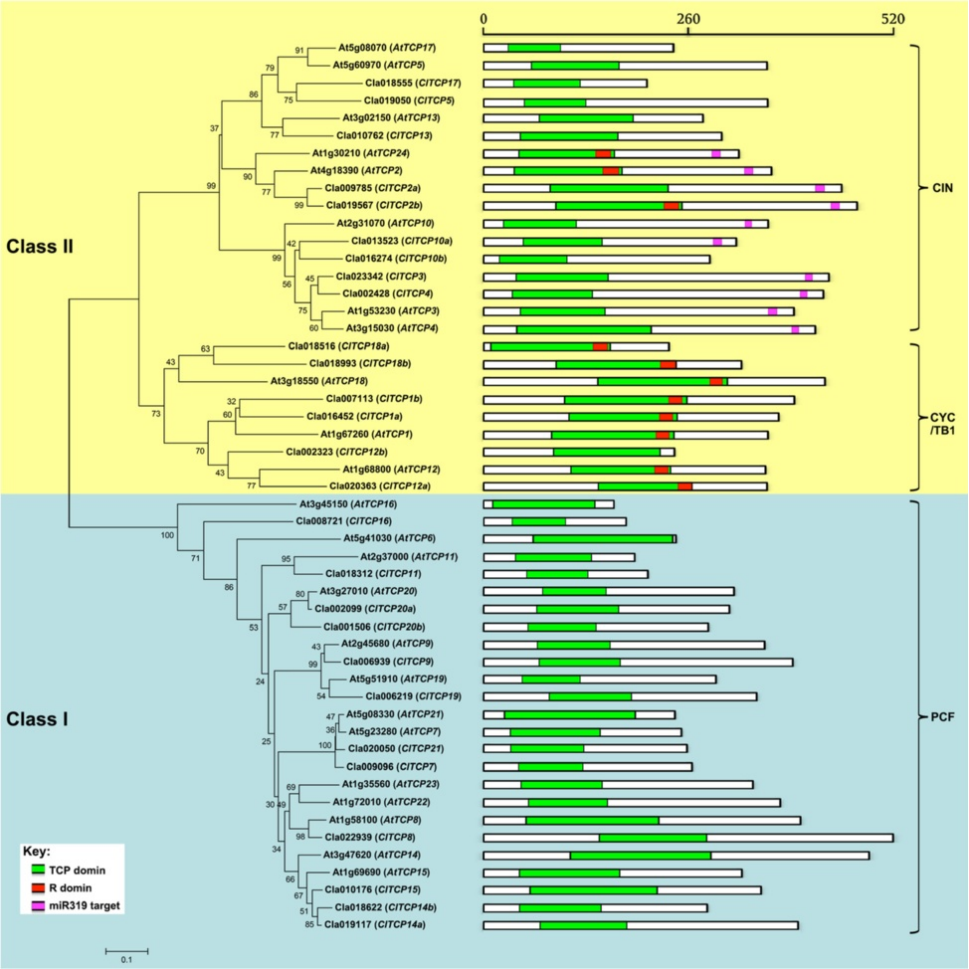
Phylogenetic analysis and conserved motifs of TCP family members in *Arabidopsis thaliana* and *Citrullus lanatus*. An unrooted phylogenetic tree, showing relationships between all TCP transcription factors in *A. thaliana* (At) and *C. lanatus* (Cl), was constructed using MEGA 5.0 with the neighbor-joining method. Support for the topology was assessed by means of a bootstrap analysis with 1000 replicates. Class I is highlighted in blue, and Class II is highlighted in yellow. On the right is the protein structure constructed using DOG 2.0 indicating conserved motifs: TCP domain (green) (http://pfam.xfam.org), R domain (red) (PlntTFDB database). The position of the microRNA miR319 recognition sequence in the mRNA is indicated in light purple (not drawn to scale). The scale bar represents amino acid length

Analysis of the conserved motif structure was performed to confirm the validity of the phylogenetic tree. The R domain, an 18–20 residue arginine-rich motif, is absent in all Class I proteins and is mainly present in CYC/TB1 proteins. The miR319 site is only present in a subset of the CIN-like genes (Fig. 3). In *Arabidopsis*, miR319 modulates jasmonate biosynthesis, negatively regulates leaf growth, positively regulates leaf senescence and affects petal development. These functions are dependent on post-transcriptional regulation of the miR319-targeted TCP genes (*AtTCP2*, *AtTCP3*, *AtTCP4*, *AtTCP10* and *AtTCP24*) [2, 5, 27, 43]. In the present study, five CIN-type *ClTCP* genes (*ClTCP2a*, *2b*, *3*, *4* and *10a*) contained the putative miR319 target site and shared the highest sequence similarity with the *Arabidopsis* miR319-targeted TCP genes (Fig. 3). These findings indicated that regulation of hormone response and leaf development by miRNA-targeted homologous TCP TFs may be conserved in watermelon and *Arabidopsis*. In addition, exon/intron structure analysis showed that most of the *ClTCP* genes lacked an intron, with the exception of *ClTCP1a* and *ClTCP12b*, which contained one intron, and *ClTCP18a* contained two introns (data not shown). Interestingly, these three *ClTCP* genes belong to the CYC/TB1-type subclade.

### Expression profiles of TCP genes in *Citrullus lanatus*

**Fig. 4:**
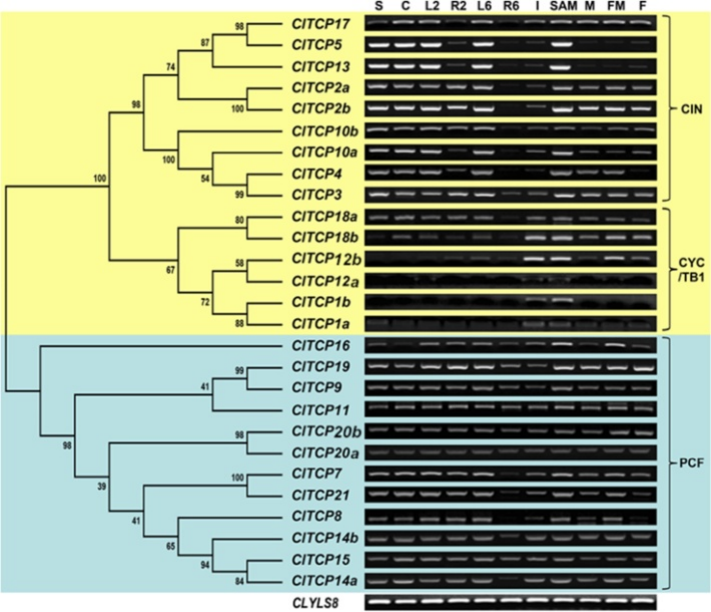
Expression patterns of *Citrullus lanatus* TCP genes in different tissues. The expression profile of *ClTCP* genes in the seed, leaf, internode, shoot tip, root, flowers and fruit was obtained through semi-quantitative PCR analysis. Expression of the *CLYLS8* gene was monitored as an internal control. The phylogenetic tree of all TCP transcription factors in *Citrullus lanatus* was constructed using MEGA 5.0 with the neighbor-joining method. Support for the topology was assessed by means of a bootstrap analysis with 1000 replicates. S: Seeds at germination; C: cotyledons; L2: leaves at two-true-leaf stage; R2: roots at two-true-leaf stage; L6: leaves at six-true-leaf stage; R6: roots at six-true-leaf stage; I: internodes; SAM: shoot apical meristem; M: male flower; FM: female flower; F: immature fruit 3 hours after pollination

To predict possible functions of TCP genes in watermelon, we performed semi-quantitative PCR (semi-qPCR) analysis of transcripts in different organs, including the seed, cotyledon, leaf, root, internode, shoot apical meristem (SAM), male and female flower buds, and fruit. Interestingly,expression analysis showed that every class/clade showed a characteristic expression profile. As indicated in Fig. 4, most CIN-type *ClTCP* genes were not expressed or only weakly expressed in the root, flower or fruit, and were more highly expressed in the seed, cotyledon and leaf, which suggested that these genes may perform important roles in the shoot. Most CYC/TB1-type *ClTCP* genes were relatively weakly expressed in the seed, leaf of early stage and root, but were relatively highly expressed in specific tissues. For example, *ClTCP18b* and *ClTCP12b* were relatively highly expressed in the internode and SAM beyond the six-leaf stage, and in the flower and fruit, whereas *ClTCP1a, ClTCP1b* and *ClTCP12a* were only expressed in the internode and SAM beyond the six-leaf stage. These results indicated that CYC/TB1-type *ClTCP* genes might play important roles in the development of internodes and flowers. Generally, Class II TCP genes, which function in a similar manner mainly by suppressing cell division and plant growth, exhibit tissue-specific expression pattern. CYC/TB1 subclade genes have been long considered to be key players in the development of axillary meristems giving rise to either flowers or lateral shoots. *AtTCP1*, the gene most closely related to *CYC*, is involved in the longitudinal elongation of leaves. The *Arabidopsis* gain-of-function *tcp1-1D* mutant shows an elongated-leaf phenotype, whereas expression of a *TCP1-SRDX* chimeric repressor gene in the wild type results in the opposite phenotype to the *tcp1-1D* mutant [9, 40]. Moreover, mutation of the *HaCYC2c* gene, a *TCP1/CYC* homolog in sunflower, promoted the developmental switch from sterile to hermaphrodite flowers [44]. Expression of At*TCP1* is strong in the petiole, lower portion of the inflorescence stem, and the midrib and distal region of expanding rosette leaves. Two *ClTCP1* genes, which are closely related to *AtTCP1*, were strongly expressed in the internode and SAM of watermelon (Fig. 4). This result is partly consistent with the expression pattern of *AtTCP1* in *Arabidopsis* and implies that *ClTCP1* genes may play roles in internode and inflorescence development in watermelon. *AtTCP18*, which is also known as *BRANCHED1* (*BRC1*) and *TEOSINTE BRANCHED1-LIKE1* (*TBL1*), acts downstream of auxin and strigolactone to coordinate axillary bud outgrowth [4, 36]. *AtTCP18* also represses the floral transition of the axillary meristems by interacting with *FLOWERING LOCUS T* (*FT*) [45]. *AtTCP12*, also known as *BRC2*, exhibits a weaker or no mutant phenotype compared with *AtTCP18* [4, 36]. Furthermore, no interactions between the BRC2 and FT proteins have been detected in yeast two-hybrid experiments [45]. In watermelon, the expression level of *ClTCP12b* and *ClTCP18b* was significantly higher in the internode, SAM, flower and fruit. Expression of *ClTCP12a* was detected only in the internode and SAM (Fig. 4). These observations suggested that these genes are likely to perform similar roles in branch and/or inflorescence development in watermelon to those of the *Arabidopsis* homologs. In contrast, CIN-type TCP genes are considered to have originated prior to CYC/TB1-type TCPs and are important for generation of the flat surface and smooth margin of the leaf. Thus, *cin*-type mutants usually exhibit crinkly and/or serrated leaves [2, 23, 27, 35]. In watermelon, expression of all CIN-type TCP genes was detected in the cotyledon, leaf and SAM (Fig. 4). This result is consistent with their predicted roles in leaf and lateral-organ development.

In contrast, most Class I genes, which usually promote plant growth and cell proliferation, showed more widespread and less tissue-specific expression patterns, such as in leaf, flower, and at an early stage of fruit development (Fig. 4). This finding implied that these genes may play diverse regulatory roles at multiple development stages. In *Arabidopsis*, several important functions of Class I TCP TFs have been discovered even though few phenotypic variations are observed in the single mutants. For example: *AtTCP8* is proposed to be involved in mitochondrial biogenesis [46]. *AtTCP14* and *AtTCP15* are reported to modulate cell proliferation during seed, leaf, floral and internode development [31, 33, 47]. *AtTCP15* may also be important for endoreduplication [48]. *AtTCP16* plays a role in early pollen development [3]. *AtTCP20*, which acts upstream of *AtTCP9*, controls leaf development via the jasmonate signaling pathway [13, 15, 28]. All of these AtTCP genes have at least one counterpart in watermelon, implying that Class I TCP in watermelon may perform similar functions. Taken together, the above-mentioned findings from model plants highlight that the TCP family performs diverse functions in multiple biological processes. *ClTCP* genes are likely to share conserved functions with *Arabidopsis* homologs, as they show not only high sequence similarity but also similar expression patterns.

### Role of *ClTCP14a* and *ClTCP15* in plant height

**Fig. 5:**
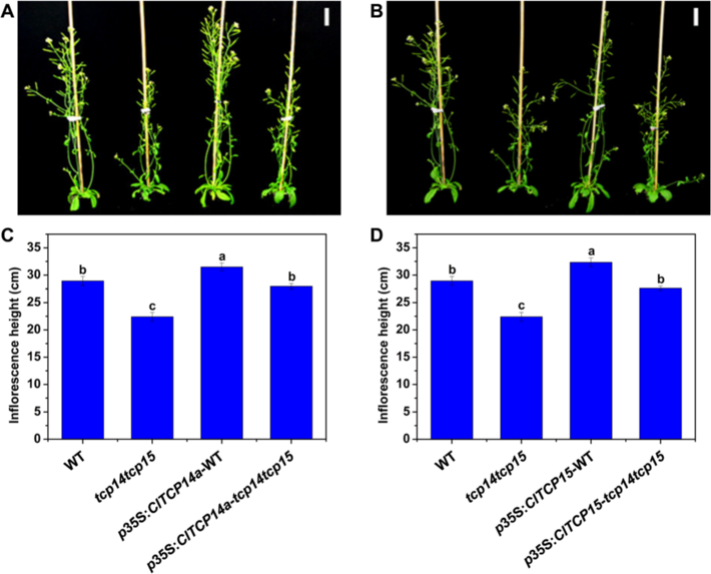
Morphological effects of constitutive expression of *ClTCP14a* and *ClTCP15* in transgenic *Arabidopsis*. **A** Seedlings of the wild type (WT; Col-0), double mutant (*tcp14 tcp15*) and *p35S:ClTCP14a* in WT and double-mutant backgrounds were grown under long-day conditions for 42 days. **B** Seedlings of the WT, double mutant and *p35S:ClTCP15* in WT and double-mutant backgrounds were grown under long-day conditions for 42 days. **C** Inflorescence height of seedlings as shown in (**A**). **D** Inflorescence height of seedlings as shown in (**B**). Scale bars = 3 cm. Different lower-case letters denote a significant difference in inflorescence height among genotypes (*P* < 0.05, one-way ANOVA and then Tukey’s test for multiple comparisons). Values are means ± SD (*n* = 20)

*ClTCP14a* and *ClTCP15* are members of the Class I subfamily of TCP TFs in watermelon (Fig. 3). These two ClTCP genes are closely related to *Arabidopsis AtTCP14* and *AtTCP15* as well as *Antirrhinum* TCP TF *TIC* [33, 49]. Given the unavailability of a *TCP*-related mutant in watermelon, we examined the function of these two *ClTCP* genes in four independent transgenic lines (*p35S:ClTCP14a*-WT, *p35S:ClTCP15*-WT, *p35S:ClTCP14a-tcp14 tcp15* and *p35S:ClTCP15-tcp14 tcp15*), which over-expressed *ClTCP14a* or *ClTCP15* in both *Arabidopsis* Col-0 and *tcp14 tcp15* double-mutant backgrounds. After growth under long-day conditions for 42 days, the double-mutant seedlings showed a significant reduction in inflorescence height than that of the wild type (Fig. 5). No visible phenotype was identified in any single mutant, similar to the observations of Kieffer et al. [33]. Ectopic expression of either *ClTCP14a* or *ClTCP15* was sufficient to restore the inflorescence height and stem internodes length of *tcp14 tcp15* double mutant to that of the wild type. The *p35S:ClTCP14a*-WT and *p35S:ClTCP15*-WT lines exhibited an increase in inflorescence height compared with that of the wild type (Fig. 5). These results suggested that *ClTCP14a* and *ClTCP15* function redundantly to control *Arabidopsis* plant height and may play positive roles in stem internode elongation in watermelon. Scanning electron microscopy revealed that the double mutant bore excessively branched trichomes compared with those of the wild type, and that overexpression of *ClTCP14a* or *ClTCP15* inhibited trichome branching in both backgrounds (Additional file 4: Figure S3). Furthermore, ectopic expression of each watermelon TCP gene in both backgrounds increased the relative chlorophyll content in mature leaves (Additional file 5: Figure S4). These findings suggested that *ClTCP14a* and *ClTCP15* may also be involved in leaf development.

**Fig. 6:**
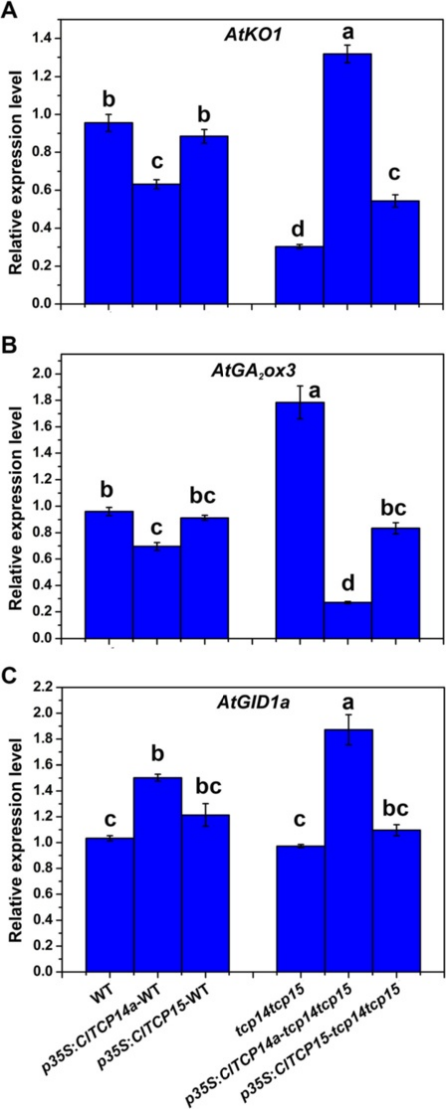
Expression of gibberellic acid (GA)-related genes in transgenic *Arabidopsis*. The relative expression level of **A** the GA biosynthesis gene *AtKO1*, **B** the GA degradation gene *AtGA*_*2*_ *ox3* and **C** the GA receptor gene *AtGID1a* in seedlings of the wild type (WT), double-mutant (*tcp14 tcp15*), *p35S:ClTCP14a* and *p35S:ClTCP15* in both WT and double-mutant backgrounds was determined by quantitative RT-PCR. Expression of the *CLYLS8* gene was monitored as an internal control. Different lower-case letters denote a significant difference in relative expression level (*P* < 0.05, one-way ANOVA and then Tukey’s test for multiple comparisons). Values are means ± SD (*n* = 3)

Given that GA is a regulator of plant height, we investigated whether overexpression of *ClTCP14a* and *ClTCP15* affected GA biosynthesis and metabolism. *AtKO1* and *AtGA*_*2*_*ox3*, which are involved in GA biosynthesis and degradation, were more weakly and more highly expressed, respectively, in the *tcp14 tcp15* double mutant compared with those of the wild type. Ectopic expression of each watermelon *TCP* in the *tcp14 tcp15* background revealed positive and negative impacts on the expression of *AtKO1* and *AtGA*_*2*_*ox3*, respectively (Fig. 6A and B). However, these effects were not observed in the wild-type background. The GA receptor, *AtGID1a*, was slightly but significantly up-regulated in *ClTCP14a-* and *ClTCP15-* overexpressing *Arabidopsis* (Fig. 6C). These results suggested that overexpression of *ClTCP14a* and *ClTCP15* may enhance GA accumulation and signaling in *tcp14 tcp15* and the effects of these TCP genes on plant height may be associated with the GA pathway. Interestingly, the expression of all GA-related genes differed significantly between *ClTCP14a*- and *ClTCP15*-transgenic *Arabidopsis*, which might reflect the higher expression level of *ClTCP14a* compared with that of *ClTCP15* in each transgenic line (Additional file 6: Figure S5).

**Fig. 7:**
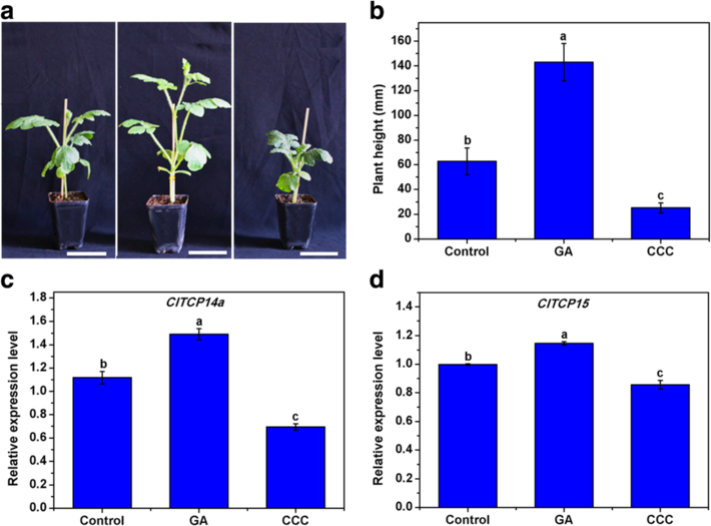
Effects of gibberellic acid (GA) and chlormequat chloride (CCC) on plant height and expression of *ClTCP* genes in watermelon. Watermelon seedlings were treated with water (Control), GA and CCC at the two-true-leaf stage. **a** and **b** Plant height of watermelon seedlings at the six-true-leaf stage. Scale bars = 7 cm. Different lower-case letters denote a significant difference in plant height between treatments (*P* < 0.05, one-way ANOVA and then Tukey’s test for multiple comparisons). Values are means (*n* = 5) ± SD. **c** Relative expression level of *ClTCP14a* in watermelon seedlings 1 day after treatment. **d** Relative expression level of *ClTCP15* in watermelon seedlings 1 day after different treatments. Different lower-case letters denote a significant difference in relative expression level (*P* < 0.05, one-way ANOVA and then Tukey’s test for multiple comparisons). Values are means ± SD (*n* = 3)

In addition, the effects of GA and chlormequat chloride (CCC, a GA biosynthesis inhibitor) on plant height as well as *ClTCP14a* and *ClTCP15* expression were examined in watermelon. The results revealed that GA and CCC were a functional enhancer and inhibitor, respectively, of watermelon plant height (Fig. 7a and b). Both regulators likely function by affecting internode length rather than internode number, as no differences in internode numbers were observed. Expressions of both *ClTCP14a* and *ClTCP15* was significantly up- and down-regulated by GA and CCC treatment, respectively (Fig. 7c and d). These results confirmed that *ClTCP14a* and *ClTCP15* might positively regulate watermelon plant height and internode length via a GA-related pathway.

Plant height is an important agronomic trait in watermelon, which dramatically affects planting density and fruiting position in the field. TCP TFs, as well known cell proliferation regulators, are undoubtedly important participants in internode and plant elongation. The present results revealed that *ClTCP14a* and *ClTCP15* redundantly regulated internode length and plant height via a GA-related pathway in transgenic *Arabidopsis* (Figs. 5, 6 and 7). In *Arabidopsis*, *AtTCP14* and *AtTCP15* are reported to regulate internode development by promoting cell proliferation, based mainly on the phenotypes observed in double-mutant and *TCP14:SRDX* lines [33]. The present results provide direct evidence for this genotype–phenotype correlation. Moreover, *AtTCP14* and *AtTCP15* are expressed in internodes of young inflorescence stems, young flower pedicels, cotyledons and leaf primordia [33]. These results are generally consistent with the present expression analysis of *ClTCP* genes in watermelon (Fig. 4). Moreover, it was reported recently that *AtTCP14* and *AtTCP15* mediate GA-dependent activation of the cell cycle during seed germination [31]. Thus, we hypothesize that ClTCP14a and ClTCP15 may also act downstream of GA and promote cell proliferation during internode formation in a similar manner. Interestingly, our findings suggest that ClTCP14a and ClTCP15 might also affect GA biosynthesis and signaling (Fig. 6), which might result from a feedback regulatory mechanism.

## Conclusions

In this study, 27 TCP genes were identified in the watermelon genome, which were distributed on nine chromosomes with different densities. These TCP genes were classifiable into two classes based on the similarity in TCP domain. Expression analysis showed that members of each class/clade show a similar expression pattern. Moreover, many *ClTCP* genes showed a similar expression pattern to that of their *Arabidopsis* homologs, which suggests that the TCP family shows conserved functions in the two species. In addition, the function of two *ClTCP* genes, *ClTCP14a* and *ClTCP15*, in the regulation of internode elongation was confirmed. Ultimately, these findings will lead to potential applications for the improvement of watermelon cultivars via genetic engineering.

## Methods

### Plant materials and growth conditions

Watermelon (*Citrullus lanatus* L. cv. IVSM9, an inbred line developed by the Laboratory of Germplasm Innovation and Molecular Breeding, Zhejiang University) was used as the main plant material. Plants were grown under a photoperiod of 16 h at 27 °C (day) and 8 h at 24 °C (night) in a phytotron with a photosynthetic photon flux density of 600 μmol m^−2^ s^−1^ and relative humidity of 70–80 %.

*Arabidopsis thaliana* ecotype Columbia-0 (Col-0) was used as the wild type. All *Arabidopsis* materials, including *tcp14-4*, *tcp15-3*, *tcp14-4 tcp15-3* and their background were obtained from the University of Leeds, UK, and were genotyped by PCR as described by Kieffer et al. [33]. Plants were grown in Sanyo growth chambers (Sanyo, http://www.sanyobiomedical. co.uk) at 20 °C under long-day conditions with a photoperiod of 16 h/8 h (day/night), photosynthetic photon flux density of 200 μmol m^−2^ s^−1^ and 60 % relative humidity.

### Chromosomal analysis

Information on the chromosomal locations of all *AtTCP* genes was obtained from The Arabidopsis Information Resource (TAIR; http://www.arabidopsis.org), and that for all *ClTCP* genes was obtained through BLASTN searches against the Cucurbit Genomics Database (http://www.icugi.org). All TCP genomic data were visualized in a circos map using CIRCOS software (http://circos.ca).

### Sequence alignment and phylogenetic analysis

The sequences of 24 TCP family members in the genome of *Arabidopsis* were retrieved from TAIR (http://www.arabidopsis.org) or PlantTFDB (http://planttfdb.cbi.pku.edu.cn/). Twenty-seven *ClTCP* genes were identified from a BLAST analysis of the Cucurbit Genomics Database (http://www.icugi.org). A multiple sequence alignments of the amino acid sequences of the TCP proteins of *Citrullus lanatus* and *Arabidopsis* was generated with ClustalX 2.0 software with the default settings as described by Thompson et al. [50]. An unrooted phylogenetic tree based on the sequence alignments was constructed using MEGA 5.0 software (http://www.megasoftware.net/) [51] and the neighbor-joining method with the following parameters: pairwise alignment, 1000 bootstrap replicates, Poisson correction model, uniform substitution rates and complete deletion. In addition, a separate phylogenetic tree was constructed for all of the TCP protein sequences from *Citrullus lanatus* for further analysis.

### Identification of conserved motifs

AtTCP and ClTCP protein sequences were submitted to online searches with the Pfam (http://pfam.xfam.org) and SMART (http://smart.embl-heidelberg.de) tools to identify conserved TCP domains. The R domain was obtained from PlantTFDB (http://planttfdb.cbi.pku.edu.cn/). The method of identifying miR319-targeting TCP genes was described previously [2]. To visualize protein domain structures, IBS 1.0 software (http://www.mybiosoftware.com/ibs-illustrator-of-biological-sequences.html) was used.

### RNA isolation and RT-PCR analysis

Total RNA was isolated from tissues using the RNAprep Pure Plant Kit and treated with DNase I (Tiangen, http://www.tiangen.com). RNA concentration and quality were assessed using a Thermo 2000 Bioanalyzer with a RNA NanoDrop (Thermo Scientific, http://www.thermo.com). Reverse transcription was performed with 1 μg total RNA in a 20-μl volume, using the ReverTra Ace® qPCR RT Master Mix with gDNA Remover kit (Toyobo) and diluted to 200 μl with water. For semi-qPCR and PCR, 1 μL reverse-transcription product was used as the PCR template in a 20-μl volume reaction. Different PCR annealing temperatures were applied to optimize results and the PCR was terminated after 35 cycles. PCR products were separated by 1.5 % agarose gel electrophoresis and visualized under an ultraviolet scanner. For RT-qPCR analysis, a 20-μl qPCR mixture was employed, which contained 2.5 μl first-strand cDNAs, 10 μl 2× FastStart Universal SYBR Green Master (Roche) and 0.25 μM of the forward and reverse primers for each gene. Relative expression levels of each gene were normalized to mRNA levels of yellow-leaf-specific protein 8 (CLYLS8) as a loading control. Three biological replicates were analyzed in each case. CT values were obtained with the Real-Time PCR System StepOne version 2.1 software (Applied Biosystems). Relative fold expression changes were calculated by the comparative CT method: fold change was calculated as 2^−∆∆CT^. The ∆CT values were calculated as the difference between the CT value and the CT value of CLYLS8. ∆∆CT was the difference between the ∆CT value of TCP genes and the ∆CT value of the reference gene. The gene-specific primers for semi-quantitative PCR and RT-qPCR procedures are listed in Additional file 7: Table S2.

### Vector constructs

To study the function of *ClTCP14a* and *ClTCP15*, two constructs were developed using the *CaMV35S* promoter. Full-length *ClTCP14a* cDNA (1197 bp) and *ClTCP15* cDNA (1062 bp) were amplified by two-round PCR: the first round with the gene-specific primers *ClTCP14a*-Fl-F and *ClTCP14a*-Fl-R, and *ClTCP15*-Fl-F and *ClTCP15*-Fl-R, respectively; the second round with the common primers attB1-F and attB2-R. Finally, both sequences were cloned into the Gateway™ vector pMDC83 (Invitrogen) via the BP and LR reactions as described by Curtis and Grossniklaus [52]. The primers used for vector construction are listed in Additional file 8: Table S3.

### Transformation of *Arabidopsis*

The two constructs were transformed into *Agrobacterium tumefaciens* strain GV3101 (pMP90) [53]. Transformation of both wild-type and *tcp14 tcp15* plants was conducted by means of the floral dip method [54]. After transformation, plants were kept in a growth chamber until seed set. Transformant selection was done on germination medium containing 50 μg ml^−1^ Hygromycin-B (Roche, http://www.roche.com) for 10 days, after which germinated T_1_ seedlings were transferred to soil and grown until seed set. During selection of T_1_ plants, a plant line negative for hygromycin resistance was selected and maintained as a negative control. In addition, overexpression analysis of *TCP14a* or *TCP15* in candidate transgenic *Arabidopsis* was employed to confirm the successful transformation (Additional file 6: Figure S5). Homozygous T_3_ plants were used in this study. Sixty primary transformants were identified and analyzed in most experiments.

### Internode measurement

Inflorescence height and internode length of 42-day-old *Arabidopsis* plants were measured. All measurements were obtained from three independent experiments, and at least 10 replicate seedlings were measured in each experiment.

### Microscopy

Samples dissected and prepared for scanning electron microscopic analysis were analyzed as previously described [33].

### Phytohormone treatment

Gibberellic acid (150 mg L^−1^) (Biotech Grade biosharp, http://www.biosharp.cn) and 150 mg L^−1^ chlormequat chloride (Shanghai BioRc Co., Ltd.) were sprayed onto the leaf surface of watermelon seedlings at the two-true-leaf stage. Water was applied as the control. Three days later, the treatment was repeated. The plant height was measured at the six-true-leaf stage.

### Availability of supporting data

The datasets supporting the results of this article are available at http://dx.doi.org/10.5061/dryad.9pp6q

## Additional files

Additional file 1: Figure S1. Alignment of the predicted amino acid sequences for members of the watermelon TCP family. (PDF 3445 kb) Additional file 2: Table S1. Orthologous relationship of TCPs between watermelon and *Arabidopsis*. (XLSX 39 kb) Additional file 3: Figure S2. Phylogenetic relationships of TCP transcription factors from watermelon, *Arabidopsis*, rice and sorghum. (PDF 2881 kb) Additional file 4: Figure S3. Effect of *ClTCP14a* and *ClTCP15* on trichome branching on *Arabidopsis* leaf. (TIF 3851 kb) Additional file 5: Figure S4. Relative chlorophyll content in *Arabidopsis* leaves. (PDF 371 kb) Additional file 6: Figure S5.
*ClTCP14a* and *ClTCP15* overexpression level in transgenic *Arabidopsis. (PDF 893 kb)*
Additional file 7: Table S2. Gene-specific primers used for semi-qPCR and RT-qPCR. (XLSX 47 kb) Additional file 8: Table S3. Primers used for vector construction. (XLSX 30 kb)
