# Supplementary material for: Genome-wide identification and expression analysis of the ClTCP transcription factors in Citrullus lanatus
Source: BMC Plant Biol. 2016 Apr 12;16:85. doi: 10.1186/s12870-016-0765-9 (PMC4830022; doi:10.1186/s12870-016-0765-9)
Supplement: Additional file 1: Figure S1. — Alignment of the predicted amino acid sequences for members of the watermelon TCP family. (PDF 3445 kb) [file 12870_2016_765_MOESM1_ESM.pdf]

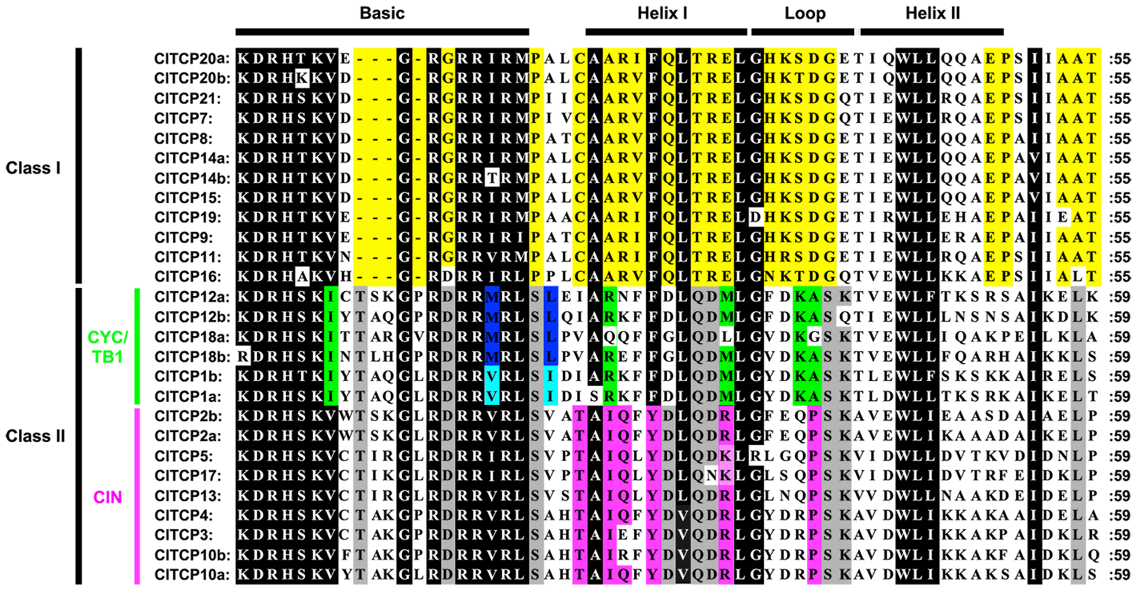

Basic
Helix I
Loop
Helix II
Class I
Class II
CYC/ TB1
CIN
CITCP20a: KDRHTKVE---G-RGRRIRMPALCAARIFQLTRELGHKSDGETIQWLLQQAEPSIIAAT :55
CITCP20b: KDRHKKVD---G-RGRRIRMPALCAARVFQLTRELGHKTDGETIQWLLQQAEPSIIAAT :55
CITCP21: KDRHSKVD---G-RGRRIRMPIICAARVFQLTRELGHKSDGQTIEWLLRQAEPSIIAAT :55
CITCP7: KDRHSKVD---G-RGRRIRMPIVCAARVFQLTRELGHKSDGQTIEWLLRQAEPSIIAAT :55
CITCP8: KDRHTKVD---G-RGRRIRMPATCAARVFQLTRELGHKSDGETIEWLLQQAEPAIIAAT :55
CITCP14a: KDRHTKVD---G-RGRRIRMPALCAARVFQLTRELGHKSDGETIEWLLQQAEPAVIAAT :55
CITCP14b: KDRHTKVD---G-RGRRTRMPALCAARVFQLTRELGHKSDGETIEWLLQQAEPAVIAAT :55
CITCP15: KDRHTKVD---G-RGRRIRMPALCAARVFQLTRELGHKSDGETIEWLLQQAEPAVIAAT :55
CITCP19: KDRHTKVE---G-RGRRIRMPAACAARIFQLTRELDHKSDGETIRWLLEHAEPAIIEAT :55
CITCP9: KDRHTKVE---G-RGRRIRIPATCAARIFQLTRELGHKSDGETIRWLLERAEPAIIAAT :55
CITCP11: KDRHTKVN---G-RGRRVRMPALCAARIFQLTRELGHRSDGETIEWLLRQAEPSIIAAT :55
CITCP16: KDRHAKVH---G-RDRRIRLPPLCAARVFQLTRELGNKTDGQTVEWLLKKAEPSIIALT :55
CITCP12a: KDRHSKICTSKGPRDRRMRLSLEIARNFFDLQDMLGFDKASKTVEWLFTKSRSAIKELK :59
CITCP12b: KDRHSKIYTAQGPRDRRMRLSLQIARKFFDLQDMLGFDKASQTIEWLLLNSNSAIKDLK :59
CITCP18a: KDRHSKITTARGVRDRRMRLSLPVAQQFFGLQDLLGVDKGSKTVEWLLIQAKPEILKLA :59
CITCP18b: RDRHSKINTLHGPRDRRMRLSLPVAREFFGLQDMLGVDKASKTVEWLLFQARHAIKKLS :59
CITCP1b: KDRHTKIYTAQGLRDRRVRLSIDIARKFFDLQDMLGYDKASKTLEWLFSKSKKAIRELS :59
CITCP1a: KDRHSKIYTAQGLRDRRVRLSIDISRKFFDLQDMLGYDKASKTLDWLLTKSRKAIKELT :59
CITCP2b: KDRHSKVWTSKGLRDRRVRLSVATAIQFYDLQDRLGFEQPSKAVEWLIEAASDAIAELP :59
CITCP2a: KDRHSKVWTSKGLRDRRVRLSVATAIQFYDLQDRLGFEQPSKAVEWLIKAAADAIKELP :59
CITCP5: KDRHSKVCTIRGLRDRRIRLSVPTAIQLYDLQDKLRLGQPSKVIDWLLDVTKVDIDNLP :59
CITCP17: KDRHSKVCTIKGLRDRRIRLSVPTAIQLYDLQNKLGLSQPSKVIDWLIDVTRFEIDKLP :59
CITCP13: KDRHSKVCTIRGLRDRRVRLSVSTAIQLYDLQDRLGLNQPSKVVDWLLNAAKDEIDELP :59
CITCP4: KDRHSKVCTAKGPRDRRVRLSAHTAIQFYDVQDRLGYDRPSKAVDWLIKKAKAAIDELA :59
CITCP3: KDRHSKVCTAKGPRDRRVRLSAHTAIEFYDVQDRLGYDRPSKAVDWLIKKAKPAIDKLR :59
CITCP10b: KDRHSKVFTAKGPRDRRVRLSAHTAIRFYDVQDRLGYDRPSKAVDWLIKKAKFAIDKLQ :59
CITCP10a: KDRHSKVYTAKGLRDRRVRLSAHTAIQFYDVQDRLGYDRPSKAVDWLIKKAKSAIDKLS :59
