# Supplementary figures and images for: Genome-wide identification and expression analysis of the ClTCP transcription factors in Citrullus lanatus
Source: BMC Plant Biol. 2016 Apr 12;16:85. doi: 10.1186/s12870-016-0765-9 (PMC4830022; doi:10.1186/s12870-016-0765-9)

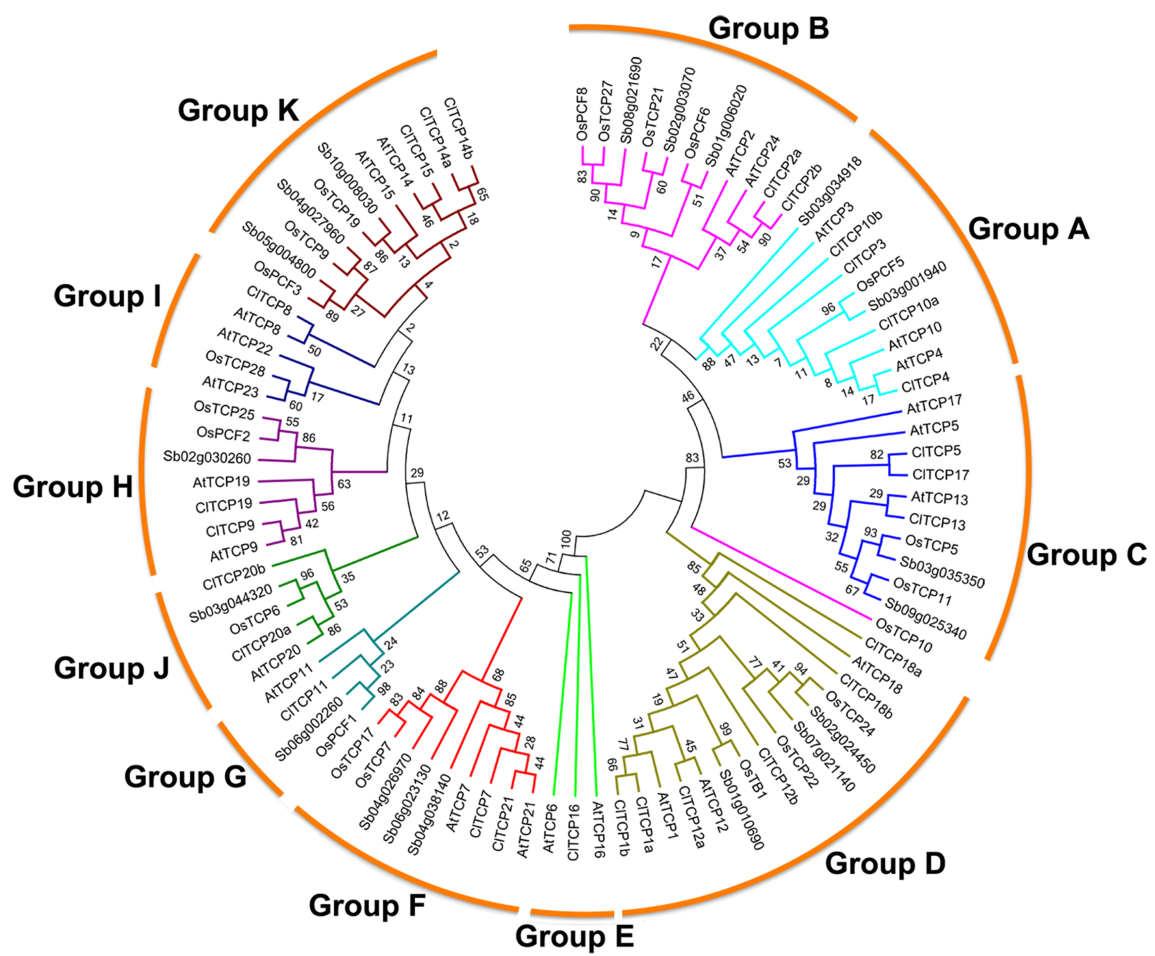

Supplement: Additional file 3: Figure S2. — Phylogenetic relationships of TCP transcription factors from watermelon, Arabidopsis, rice and sorghum. (PDF 2881 kb) [file 12870_2016_765_MOESM3_ESM.pdf]

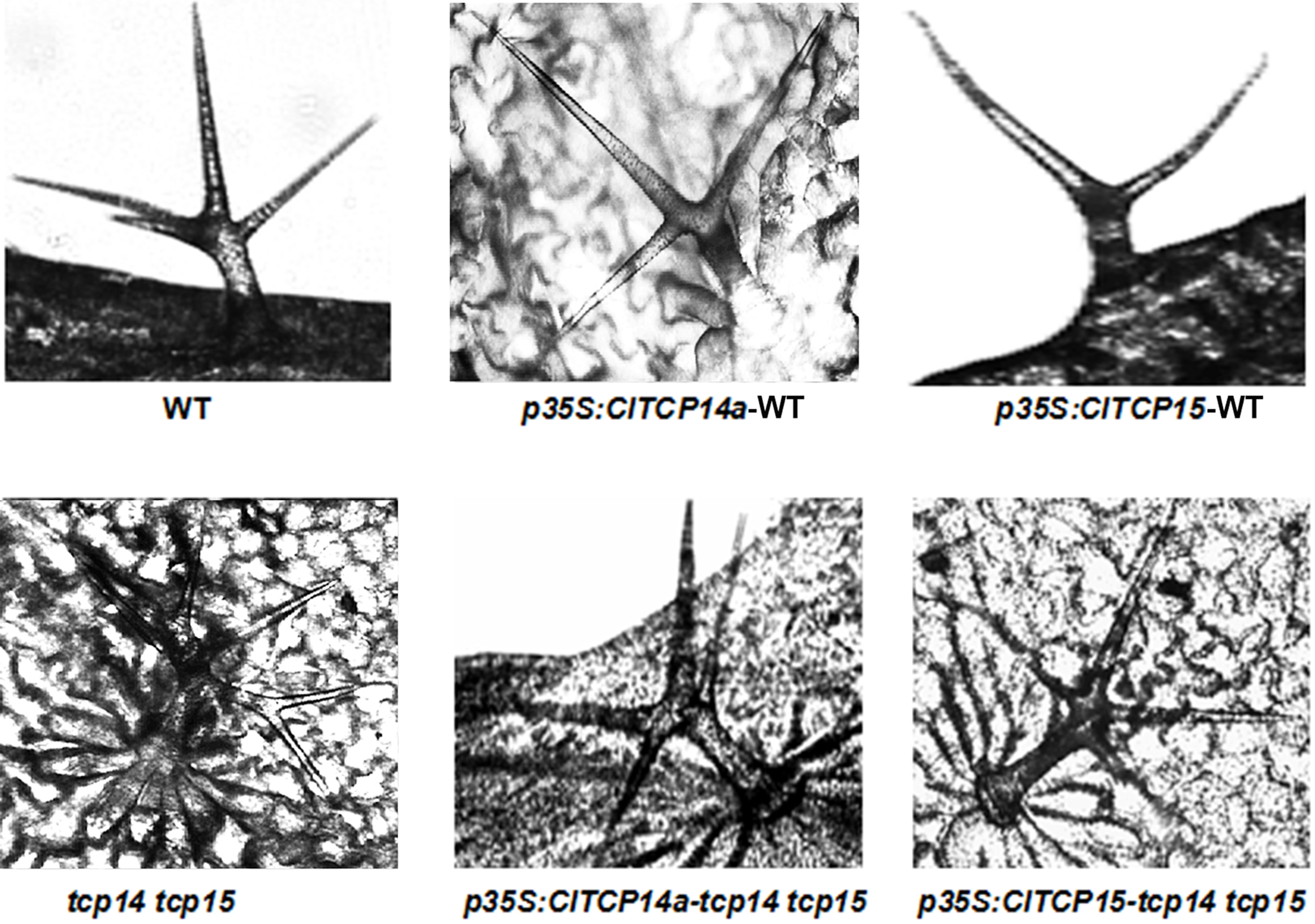

Supplement: Additional file 4: Figure S3. — Effect of ClTCP14a and ClTCP15 on trichome branching on Arabidopsis leaf. (TIF 3851 kb) [file 12870_2016_765_MOESM4_ESM.tif]

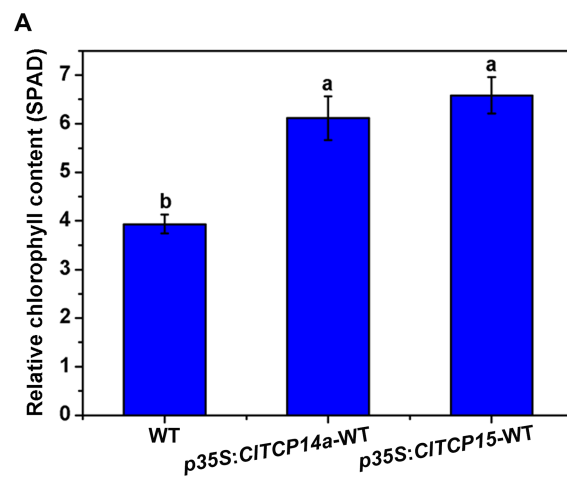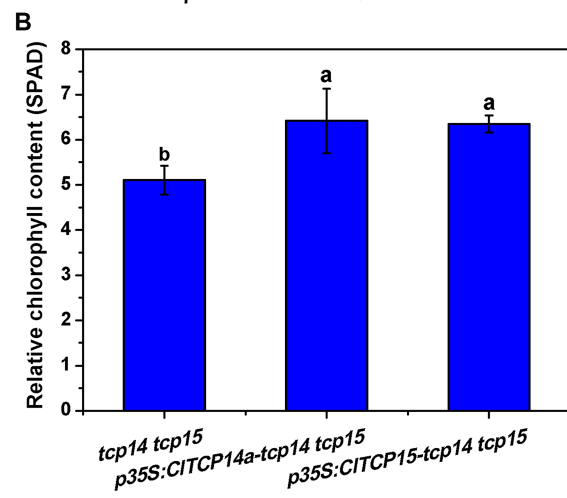

Supplement: Additional file 5: Figure S4. — Relative chlorophyll content in Arabidopsis leaves. (PDF 371 kb) [file 12870_2016_765_MOESM5_ESM.pdf]

**A**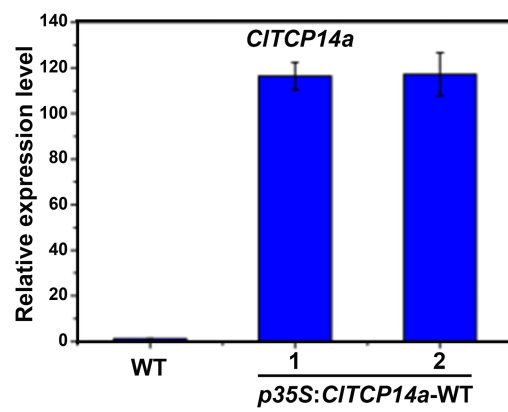**B**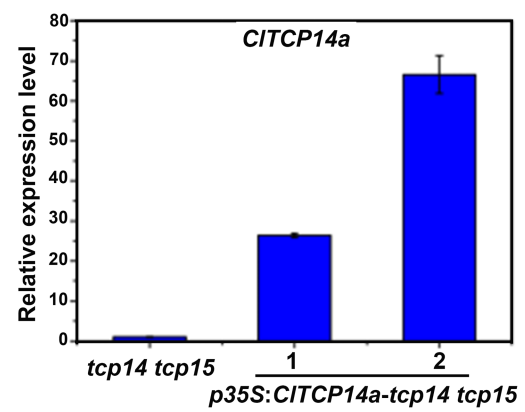**C**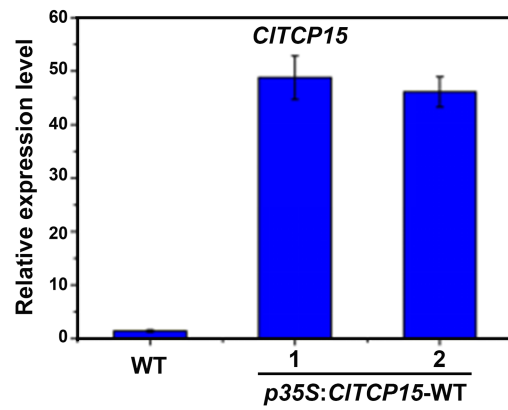**D**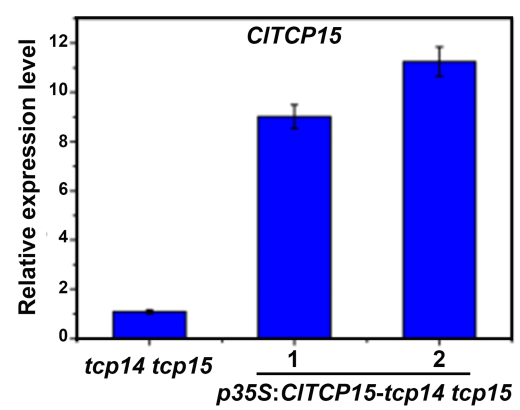

Supplement: Additional file 6: Figure S5. — ClTCP14a and ClTCP15 overexpression level in transgenic Arabidopsis. (PDF 893 kb) [file 12870_2016_765_MOESM6_ESM.pdf]
